# Atypical adverse events in a real-world study of long-term immunomodulation for multiple sclerosis and neuromyelitis optica spectrum disorder

**DOI:** 10.1177/17562864251320206

**Published:** 2025-04-04

**Authors:** Amelie Kirschbaum, Felix Luessi, Arda Civelek, Stefan Bittner, Johannes Piepgras, Frauke Zipp

**Affiliations:** Department of Neurology, Focus Program Translational Neuroscience (FTN) and Immunotherapy (FZI), Rhine-Main Neuroscience Network (rmn^2^), University Medical Center of the Johannes Gutenberg University Mainz, Mainz, Germany; Department of Neurology, Focus Program Translational Neuroscience (FTN) and Immunotherapy (FZI), Rhine-Main Neuroscience Network (rmn^2^), University Medical Center of the Johannes Gutenberg University Mainz, Langenbeckstrasse 1, Mainz 55131, Germany; Department of Neurology, Focus Program Translational Neuroscience (FTN) and Immunotherapy (FZI), Rhine-Main Neuroscience Network (rmn^2^), University Medical Center of the Johannes Gutenberg University Mainz, Mainz, Germany; Department of Neurology, Focus Program Translational Neuroscience (FTN) and Immunotherapy (FZI), Rhine-Main Neuroscience Network (rmn^2^), University Medical Center of the Johannes Gutenberg University Mainz, Mainz, Germany; Department of Neurology, Focus Program Translational Neuroscience (FTN) and Immunotherapy (FZI), Rhine-Main Neuroscience Network (rmn^2^), University Medical Center of the Johannes Gutenberg University Mainz, Mainz, Germany; Department of Neurology, Focus Program Translational Neuroscience (FTN) and Immunotherapy (FZI), Rhine-Main Neuroscience Network (rmn^2^), University Medical Center of the Johannes Gutenberg University Mainz, Mainz, Germany

**Keywords:** atypical adverse events, demyelination, disease-modifying therapy, multiple sclerosis

## Abstract

**Background:**

Immunotherapies are integral in managing multiple sclerosis (MS) and related demyelinating diseases, but adverse drug reactions significantly affect the tolerability of disease-modifying therapies (DMTs).

**Objectives:**

This study aims to assess the safety profile of DMTs within a real-world cohort affected by MS and related diseases and to identify atypical adverse events (AEs) and those of exceptional severity.

**Methods:**

A retrospective analysis was conducted on 3850 patients with MS, neuromyelitis optica spectrum disorder (NMOSD), and related conditions (2009–2022). Demographic and clinical data were analyzed for patients treated with DMTs. Parameters included prior treatments, AEs, treatment durations, and reasons for discontinuation.

**Results:**

Of the cohort, 1989 patients (71.1% female) with a median follow-up of 46.3 months during DMT use were included. Monotherapy was employed in 987 patients, while 1002 received sequential DMTs, totaling 3850 treatments. Adverse reactions led to discontinuation in 24.2% of cases, while disease progression accounted for 22.9%. Among 1878 AEs, 31 (1.7%) were atypical, and 59 (3.1%) were unusually severe, which was systematically categorized based on type, timing, and remission.

**Conclusion:**

Within the confines of this real-world study, DMT administration emerged as generally well tolerated in MS, related demyelinating diseases and NMOSD. The identification of a limited number of atypical AEs, nevertheless, broadens the spectrum of potential complications associated with DMTs. Although weaker evidence for causal associations between drug exposure and observed AEs remains a limitation in observational studies without comparable control groups, this study underscores the value of real-world investigations in offering insights into the long-term safety of DMTs, particularly for rare events.

## Introduction

Multiple sclerosis (MS) is the most common chronic inflammatory, demyelinating, and neurodegenerative disease of the central nervous system in young adults. This disorder is a heterogeneous, multifactorial, immune-mediated disease that is influenced by both genetic and environmental factors.^1^ Cardinal pathological features include multifocal inflammation, demyelination, oligodendroglial death, axonal damage, and astrocytic scarring in the brain and spinal cord.

An increasing number of immunotherapies has been approved for drug treatment over the last two decades. According to the current US Food and Drug Administration (FDA) assessment, the approved disease-modifying therapies (DMTs) encompass interferons (beta-1a, beta-1b, and peginterferon beta-1a), glatiramer acetate, anticluster of differentiation 20 (CD20) antibodies (ocrelizumab and ofatumumab), nonselective (fingolimod), or selective (siponimod, ozanimod, and ponesimod) modulators of shingosine-1-phosphate (S1P), antibodies (natalizumab and alemtuzumab), and small molecule drugs such as teriflunomide, dimethyl fumarate (DMF), and cladribine.^2^ Other immunosuppressive drugs such as azathioprine, daclizumab, and mitoxantrone are or were registered as therapy for MS. Despite still being approved as an effective therapy for highly aggressive MS, mitoxantrone is losing importance in MS therapy due to newer approved immunotherapies with strong efficacy and a more moderate side effect profile.^3^

Rapid evolution of the therapeutic landscape in MS—and only very recently in neuromyelitis optica spectrum disorder (NMOSD)—has improved the capability to minimize damage from relapses and preserve neurological function. In this study, anti-CD20 was the major therapy administered to people with NMOSD. Nevertheless, adverse drug reactions play a major role in determining the tolerability and thus the limits of applicability of individual DMTs. Researchers have increasingly delved into understanding the less common but potentially severe adverse events (AEs) associated with these DMTs. The notion that genetic and individual factors play a pivotal role in determining susceptibility to rare side effects has gained traction. The utilization of pharmacovigilance data and postmarketing surveillance has been crucial in monitoring rare side effects as DMTs enter broader clinical use. This methodology aids in the timely detection and reporting of unexpected AEs. Furthermore, the literature increasingly incorporates long-term, real-world evidence, moving beyond controlled clinical trials. This shift provides a more nuanced understanding of rare side effects that might not manifest in the relatively short durations of randomized controlled trials. Despite advancements, underreporting of rare side effects remains a challenge. Our study aims to address this gap by assessing the safety profile of DMTs within a real-world cohort affected by MS or related autoimmune disorders, in particular less-frequent chronic inflammatory NMOSD.

## Methods

### Patient cohort

**Figure 1.**
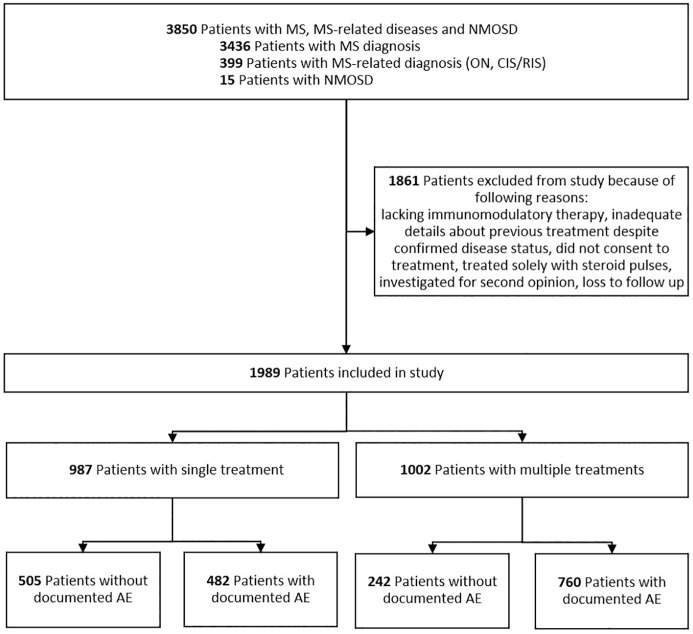
Profile of study cohort. AE, adverse event; CIS/RIS, clinically/radiologically isolated syndrome; MS, multiple sclerosis; NMOSD, neuromyelitis optica spectrum disorder; ON, optic neuritis.

We conducted a retrospective cohort investigation utilizing datasets procured from the Department of Neurology at the University Medical Center Mainz (Mainz, Germany). This study is covered by the State Hospital Act §36 and §37, and thus did not require written consent, according to the local ethics committee (Landesärztekammer Rheinland-Pfalz). The study spanned from 2009 to 2022 and encompassed all patients with suspected diagnosis of MS or NMOSD. Using an algorithmic approach, we refined the dataset to include only individuals with diagnostic information based on International Classification of Diseases, 10th Revision (ICD-10) codes for confirmed diagnoses of MS, optic neuritis, NMOSD, or clinically or radiologically isolated syndrome (CIS or RIS, respectively). Data on immunotherapies were extracted from free-text fields, such as written discharge reports and visit notes. For unusually severe or atypical side effects, additional documentation, including surgical consultations, photo documentation, laboratory results, or swabs, was reviewed to assess severity. Data extraction were performed twice: once by an experienced neurologist and a second time by a trained medical student. The steps of the data management process are shown in Supplemental Figure 1. Inclusion criteria necessitated a documented history of at least one outpatient or inpatient encounter involving DMT, fulfillment of one of the required diagnoses for study eligibility, and the availability of accurate information regarding age, sex, and prior therapeutic interventions. Conversely, patients lacking immunomodulatory therapy or exhibiting inadequate details about previous treatments, despite confirmed disease status, were excluded from the analysis (Figure 1). Moreover, patients were also excluded if they did not consent to treatment, were treated solely with steroid pulses for acute relapse, were only seen for the purpose of receiving a second opinion, or if they were lost to follow-up.

### Classification of AEs

We recorded all pharmacological interventions, along with the duration of use and the rationale underlying modifications or discontinuations. This included considerations of therapeutic inefficacy, a stable disease course, AEs of varying severity, instances of pregnancy, and less frequently encountered factors necessitating therapeutic adjustments, such as reaching the cumulative dosage threshold with mitoxantrone, patient preferences, or the increased risk of John Cunningham virus (JCV) reactivation associated with natalizumab. AEs were categorized as common, severe, or atypical as defined here.

Common AEs corresponds to AEs included in the drug information as very common (⩾1/10 patient-years) to occasional (⩾1/100 patient-years) with therapy continuation possible, therapy adjustment required but no further intervention needed, or without exceeding threshold values (e.g., laboratory-related AEs such as liver enzyme elevations exceeding five times the normal value).

Severe AEs fulfilled the FDA definition of serious AEs and were included in the drug information, but exceeding threshold values or requiring further interventions (e.g., surgical drainage of abscesses, hospitalization).^4–17^

Atypical AEs corresponds to AEs not included in the drug information requiring therapy adjustment.

Furthermore, a literature search was conducted using Pubmed (http://www.ncbi.nlm.nih.gov/pubmed) to explore observed AEs, with a specific emphasis on identifying symptoms of rarity or those not previously documented in scientific literature by using the active ingredient, agent name, and common abbreviation as search terms (“interferon beta 1a/b,” “IFN-beta1a/b,” “avonex^®^,” “rebif^®^,” “betaferon^®^,” “peginterferon beta 1a,” “pegylated IFN-beta1a,” “plegridy^®^,” “glatiramer acetate,” “copaxone^®^,” “glatopa^®^,” “dimethyl fumarate,” “BG-12,” “DMF,” “tecfidera^®^,” “sphingosine-1-phosphate receptor modulators,” “S1PM,” “fingolimod (hydrochloride),” “fty720,” “gilenya^®^,” “siponimod,” “mayzent^®^,” “ozanimod,” “zeposia^®^,” “ponesimod,” “ponvory^®^,” “anti CD20 antibody,” “ocrelizumab,” “ocrevus^®^,” “ofatumumab,” “kesimpta^®^,” “rituximab,” “RTX,” “mabthera^®^,” “teriflunomide,” “aubagio^®^,” “mitoxantrone,” “MTX,” “novantron^®^,” “ebexantron^®^,” “azathioprine,” “imurek^®^”). No extended search was conducted for active substances with no severe or atypical AEs reported (alemtuzumab, cladribine, daclizumab, methotrexate). We compared all AEs with those of existing literature.^18–38^

Complementary to the literature search, the rarely reported AEs recorded in the study population were compared with EudraVigilance (https://www.adrreports.eu), a system operated by the European Medicines Agency (EMA) to manage and analyze information on potential side effects of pharmaceuticals undergoing clinical trials or approved in the European economic area. The following search terms were used for the active ingredients (“interferon beta 1a/b,” “glatiramer acetat,” “dimethyl fumarate,” “fingolimod,” “mitoxantrone,” “azathioprine”).

### Data collection and analysis

Treatment progression and AE data were collected by an experienced neurologist and again by a trained medical student using multiple free-text fields of documented medical visits and clinician reports. No additional software or AI-based tools were used to assist with the AE data extraction process. The structure of the source medical files in the clinical information system remained unchanged throughout the study period from 2009 to 2022. All data were analyzed using Excel Office Version 365 and SPSS 27 manufacturer for Excel is Microsoft, manufacturer for SPSS 27 is IBM. Pie and line diagrams as well as sunburst charts were created using Excel Office Version 365. Quantitative variables are presented as mean values with standard error of mean. Missing data were excluded from the analyses.

A Chi-square test was conducted to assess the association between sex and the use of a specific treatment. A *p*-value less than 0.05 was defined as statistically significant.

This study was conducted in accordance with the STROBE guidelines; the completed STROBE checklist is available as Supplemental Material.

## Results

### Cohort characteristics

A cohort of 3850 patients was selected based on confirmed diagnosis of MS (*n* = 3436) or closely related conditions, including CIS or RIS (*n* = 191), and others (*n* = 223) upon immune modulatory treatment. A total of 1989 patients (mean age ± standard error of the mean = 33.1 ± 10.7 years; 1415 (71.1%) females with age of 32.5 ± 10.5 years; 574 (28.9%) males with age of 34.5 ± 11.1 years) met the stringent inclusion criteria for this study. Taking into consideration initial treatment and switching to other DMTs, this cohort experienced a combined 3850 treatment initiations (Table 1). Analysis of these treatment initiations revealed a sex distribution skewed toward females (71.5% vs 28.5%) and a mean age of 34.6 ± 0.3 years at treatment initiation, exhibiting variations among different drug groups, ranging from 26.6 ± 2.6 years for alemtuzumab to 45.3 ± 1.2 years for “other immunomodulators” which here is consisting of the historical agents mitoxantrone, methotrexate, daclizumab, or azathioprine. In the examination of 1989 patients with distinct DMTs, the average number of therapies per patient was 1.94 DMTs, with 49.6% of patients having only ever received a singular therapeutic intervention. Notably, 50.4% of patients had received different DMTs during their disease course, leading to a total of 2449 therapy changes or discontinuations subjected to evaluation in this study.

**Table 1.** Overview of treatment initiations.

Characteristic	Total	IFN	GA	DMF	S1PM	aCD20	NAT	TER	AL	CdA	Others
Number of DMT	3850	1169	646	671	335	215	281	170	17	13	333
Average age at first initiation (years)	34.6 ± 0.3	32.5 ± 0.4	35.1 ± 0.5	33.4 ± 0.6	34.4 ± 1.6	40.9 ± 1.9	30.5 ± 1.4	42.2 ± 1.7	26.6 ± 2.6	41 ± 0	45.3 ± 1.2
Average age at drug intake (years)	36.1 ± 0.2	33.3 ± 0.4	35.6 ± 0.4	35.1 ± 0.4	38.3 ± 0.6	38.8 ± 0.8	32.2 ± 0.6	42.6 ± 0.9	32.9 ± 2.0	36.0 ± 2.5	44.1 ± 0.7
Documented treatment time (years)	14501	5182	2527	2169	1242	606	937	467	50	22	1299
Average duration of intake (years)	4.4 ± 0.1	5.7 ± 0.2	4.8 ± 0.2	3.4 ± 0.1	4.0 ± 0.2	3.0 ± 0.2	3.5 ± 0.2	2.9 ± 0.2	3.6 ± 0.6	1.9 ± 0.4	5.1 ± 0.3
Gender-specific distribution of DMT (%)											
In females	2752 (71.5)	872 (74.6)	487 (75.4)	488 (72.7)	230 (68.7)	127 (59.1)	199 (70.8)	120 (70.6)	9 (52.9)	8 (61.5)	212 (63.7)
In males	1098 (28.5)	297 (25.4)	159 (24.6)	183 (27.3)	105 (31.3)	88 (41.9)	82 (29.2)	50 (29.4)	8 (47.1)	5 (39.5)	121 (36.3)
Treatment											
Initial	1989	903	418	301	52	57	53	55	5	1	144
Single	987	336	206	201	30	51	24	30	3	1	105
Multiple	2863	833	440	470	305	164	257	140	14	12	228
Adverse event (%)											
No adverse event stated	1972 (51.2)	597 (51.1)	329 (50.9)	224 (33.4)	144 (43.0)	134 (62.3)	209 (74.4)	79 (46.5)	10 (58.8)	9 (69.2)	237 (71.2)
Any adverse event stated	1878 (48.8)	572 (48.9)	317 (49.1)	447 (66.6)	191 (57.0)	81 (37.7)	72 (25.6)	91 (53.5)	7 (41.1)	4 (30.8)	96 (28.8)
Continuation of treatment	1401	284	186	336	162	179	90	69	7	8	80
Discontinuation of treatment	2449	885	460	335	173	36	191	101	10	5	253
Reasons for discontinuation											
Progressive disease	881	371	188	117	61	6	29	42	4	3	60
Stable disease	70	17	15	8	2	8	2	5	0	0	13
Adverse event	933	344	178	175	90	15	20	51	4	1	55
Pregnancy	102	40	27	12	5	2	16	0	0	0	0
Other reasons	302	40	15	13	10	2	113	2	1	1	105
Reason not determined	161	73	37	10	5	3	11	1	1	0	20
Discontinuation without further therapy	587	152	102	74	35	20	21	31	2	2	148
Cumulative adjustment of active ingredients	1862	732	358	259	139	17	170	70	9	3	105
Change to											
IFN	266	128	77	22	2	0	11	3	0	0	23
GA	228	155	15	21	13	0	8	8	0	0	8
DMF	370	167	109	12	23	1	35	13	1	0	9
S1PM	284	67	39	55	14	4	62	23	1	0	19
NAT	228	84	42	51	27	0	9	5	0	0	10
Anti-CD20-antibodies	158	5	15	36	41	7	27	7	7	3	10
TER	115	34	23	40	5	0	5	4	0	0	4
AL	12	0	0	2	4	0	5	0	0	0	1
CdA	12	1	2	3	2	0	1	3	0	0	0
Other—immunomodulation	189	91	36	17	8	5	7	4	0	0	21

Illustration of the drug-specific characteristics with focus on drug selection for initial and subsequent therapies and incidence of recorded adverse events (*n* = 1878). Initially continued therapies are compared with therapy changes/discontinuations, with the evaluation of different reasons and the following choice of active substance. Mechanism-specific consolidation of individual drugs into the following drug classes: IFNs (IFN-β1a/b; pegIFN-β1b); S1PM (fingolimod, siponimod, ozanimod, ponesimod); and anti-CD20-antibodies (rituximab, ocrelizumab, ofatumumab). Mitoxantrone, methotrexate, azathioprine, and daclizumab have been summarized under other immunomodulation. Data are expressed as mean ± standard error of mean or as *n* (%) where applicable.

aCD20, anti-CD20-antibodies; AL, alemtuzumab; CD20, cluster of differentiation 20; CdA, cladribine; DMF, dimethyl fumarate; DMT, disease-modifying therapyd; GA, glatiramer acetate; IFN, interferons; IFN-β1a/b, interferon beta 1a/b; NAT, natalizumab; pegIFN-β1b, pegylated interferon beta 1b; S1PM, sphingosine-1-phosphate receptor modulators; TER, teriflunomide.

### Analysis of treatment paradigms

**Figure 2.**
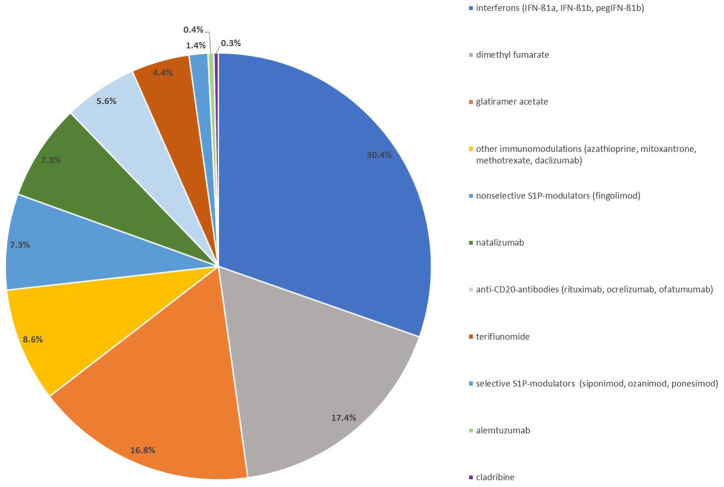
Distribution of immunomodulatory treatments in study cohort. Distribution of applied immunomodulatory treatments (*n* = 3850) in the study cohort of 1989 patients (years: 2009–2022). The division of the S1P-modulators into nonselective (fingolimod 7.3%) and selective (siponimod, ozanimod, ponesimod 1.4%) was based on their mechanisms of action. Due to a decreasing relevance in current treatment regimens, selected active substances were combined (azathioprine, methotrexate, mitoxantrone, daclizumab). CD20, cluster of differentiation 20; IFN-β1a/b, interferon beta 1a/b; MTX, methotrexate; pegIFN-β1b, pegylated interferon beta 1b; S1P-modulators, sphingosine-1-phosphate receptor modulators.

Examining the distribution of treatments with DMTs (*n* = 3850) within our cohort of 1989 patients (Table 1 and Figure 2), interferons emerged as the predominant drug class both for the initiation of primary therapy and across the spectrum of surveyed immunomodulatory interventions. However, interferons as drugs for initial treatment demonstrated adequate control of disease activity only 37.2% of the times, necessitating multiple therapeutic adjustments, including switches within or away from interferons. Glatiramer acetate and DMF ranked as the second and third most frequently used DMTs, with 49.3% and 66.8%, respectively, of initial therapies remaining unaltered throughout treatment and sustained as monotherapies. Primary reasons for altering interferons and glatiramer acetate included persistent disease activity warranting escalation.

While disease progression played a substantial role in modifying DMF therapies, adverse side effects constituted the primary impetus for alterations or discontinuations. Similar trends were observed for S1P modulators, anti-CD20 antibodies, and teriflunomide, each comprising less than 10% of all administered DMTs. Natalizumab therapy changes were primarily prompted by an elevated risk of progressive multifocal leukoencephalopathy, whereas reaching the maximum dose prompted modifications with mitoxantrone. Cladribine and alemtuzumab were least frequently utilized in our cohort (<1% of treatment initiations). The average treatment duration was 4.4 ± 0.1 years (ranging from 1.9 ± 0.4 years for cladribine to 5.7 ± 0.2 years for interferons); the primary reason for change from cladribine or alemtuzumab was disease progression.

**Figure 3.**
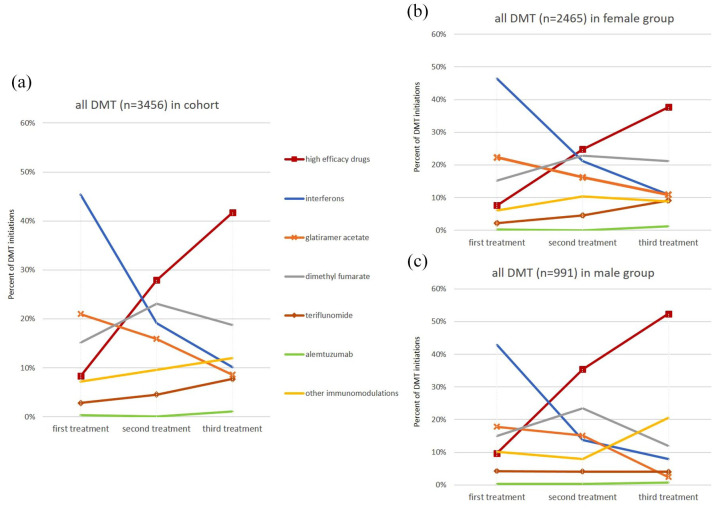
Sex-specific differences in prescription pattern of all active substances as initial, second, or third treatment. (a) Dynamics of the prescription patterns of active substances as first, second, or third treatment in our cohort. High-efficacy drugs (S1P-modulators, anti-CD20-antibodies, natalizumab, and cladribine) have been combined to obtain an overview. (b, c) Detailed examination of sex-specific differences in the prescription pattern of the treatment course of female and male patients. CD20, cluster of differentiation 20; DMTs, disease-modifying therapies; S1P-modulators, sphingosine-1-phosphate receptor modulators.

**Figure 4.**
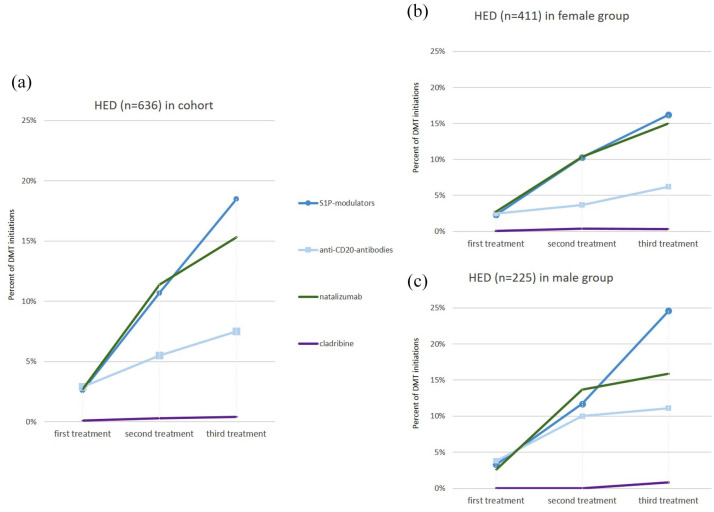
Sex-specific differences in prescription pattern in HEDs as initial, second, or third treatment. (a) Dynamics of the prescription patterns of HEDs as first, second, or third treatment in our cohort. (b, c) Detailed examination of sex-specific differences in the prescription pattern HEDs in female and male patients. HED, high-efficacy drugs.

Therapy initiation and changes to second and third DMT are depicted in Figure 3 (all DMT, *n* = 3456 treatment initiations) and Figure 4 (high-efficacy DMT only, *n* = 636 treatment initiations). Interferons and glatiramer acetate, common initial DMTs, were used less if disease activity continued during therapy (Figure 3(a)). In contrast, S1P modulators, natalizumab, and anti-CD20 antibodies (all high-efficacy drugs) assumed pivotal roles in escalation therapy (Figure 4(a)). The increased use of teriflunomide as second and third treatment observed in the female cohort (Figure 3(b)) did not significantly differ from the follow-up treatment of males (Figure 3(c); 4.6% vs 4.1%, χ^2^(1) = 0.15, *p* = 0.6985) as second treatment and 9.1% vs 4.0% %, χ^2^(1) = 3.5, *p* = 0.061 as third treatment, respectively). Alemtuzumab use was rare in our cohort. DMF featured prominently as both an initial and second-line therapy, with less frequent use as a third therapy.

Notably, sex-specific disparities were observed in prescription patterns, with high-efficacy drugs (S1P modulators, anti-CD20 antibodies, natalizumab, cladribine) administered later and less often in females compared to males, both as initial DMT (7.6% vs 9.7%, χ^2^(1) = 3.2, *p* = 0.075) and in subsequent treatments (24.8% vs 35.4%, χ^2^(1) = 9.4, *p* = 0.002 as second therapies, and 37.7% vs 52.4%, χ^2^(1) = 7.5, *p* = 0.006 as third therapies; Figure 3(b) and (c)). S1P modulators and anti-CD20 antibodies assumed increasing prominence in recent years, as evidenced by their relatively substantial proportion among subsequent DMTs (Figure 4).

In patients undergoing multiple subsequent DMTs, the predominant reasons for treatment switches or discontinuations were lack of efficacy (36.0%) and intolerable AEs (38.1%). Other contributing factors included pregnancy (4.2%) and prolonged stable disease course (2.9%). Less-frequent motivations included changes in therapy due to patient request, prioritization of therapy for another disease, and drug-specific phenomena (e.g., reaching the maximum dose of mitoxantrone or JCV seroconversion during natalizumab treatment).

Of the 2.9% of cases with a treatment switch or discontinuation due to prolonged stable disease course, 76% were female, and 24% were male. The median age at the time of treatment adjustment was 47.0 years (interquartile range (IQR): 41.2–54.7), and the median disease duration was 6.2 years (IQR: 2.5–13.1). The distribution of MS-related diagnoses was as follows: 7.7% CIS, 63.5% relapsing-remitting MS, 17.3% secondary progressive MS, and 11.5% primary progressive MS.

### Analysis of atypical AEs

Within our cohort, AE-free courses were evident in 51.2% of treatment cases, while at least one AE was recorded in 48.8%. Despite the prevalence of AE as a common reason for therapy adjustments, they were generally deemed tolerable, leading to the continuation of DMTs even in the presence of persistent symptoms (Table 2). Using the Chi-square test, we observed a significant association between the occurrence of any AEs and sex (female: 55% vs male: 48%; χ²(1) = 6.378, *p* = 0.012). In contrast, the age at treatment initiation was not significantly different between patients who did or did not experience any AEs during immunotherapy (34.30 years vs 35.38 years, *p* = 0.08).

**Table 2.** Overview of adverse drug reactions.

Groups of treatment adjustments	All drugs	IFN	GA	DMF	S1PM	aCD20	NAT	TER	AL	CdA	Others
Total therapies	3850	1169	646	671	335	215	281	170	17	13	333
No AE reported	1972	597	329	224	144	134	209	79	10	9	237
Any AE reported	1878	572	317	447	191	81	72	91	7	4	96
AE led to discontinuation (%)	933 (49.7)	344 (60.1)	178 (56.2)	175 (39.1)	90 (47.1)	15 (18.5)	20 (27.8)	51 (56.0)	4 (57.1)	1 (25.0)	55 (57.3)
AE with continuation (%)	945 (50.3)	228 (39.9)	139 (43.8)	272 (60.9)	101 (52.9)	66 (81.5)	52 (72.2)	40 (44.0)	3 (42.9)	3 (75.0)	41 (42.7)

Analysis of the incidence and consequences of adverse drug reactions (*n* = 1878) in the various drug classes regarding the continuation or necessitation of therapy adjustment in all therapies (*n* = 3850). Treatment changes or discontinuations due to other causes are not considered in this table.

aCD20, anti-CD20-antibodies; AE, adverse event; AL, alemtuzumab; CD20, cluster of differentiation 20; CdA, cladribine; DMF, dimethyl fumarate; GA, glatiramer acetate; IFN, interferons; NAT, natalizumab; S1PM, sphingosine-1-phosphate receptor modulators; TER, teriflunomide.

Notably, treatment with natalizumab exhibited the lowest proportion of AEs (in 25.6% of cases) followed by treatments with cladribine (30.8%) and anti-CD20 antibodies (37.7%). AEs were reported in 41.2% of treatments with alemtuzumab, with over half of the symptoms necessitating treatment adjustments, a proportion higher than that observed in patients treated with natalizumab (27.8%), cladribine (25.0%), or anti-CD20 antibodies (18.5%). Conversely, DMF therapies caused the highest proportion of AEs (66.6%) in our study cohort, resulting in treatment adjustment in only 39.1% of cases, while AEs occurring in nearly half of interferon treatment cases (48.9%) frequently led to discontinuation (60.1%).

**Figure 5.**
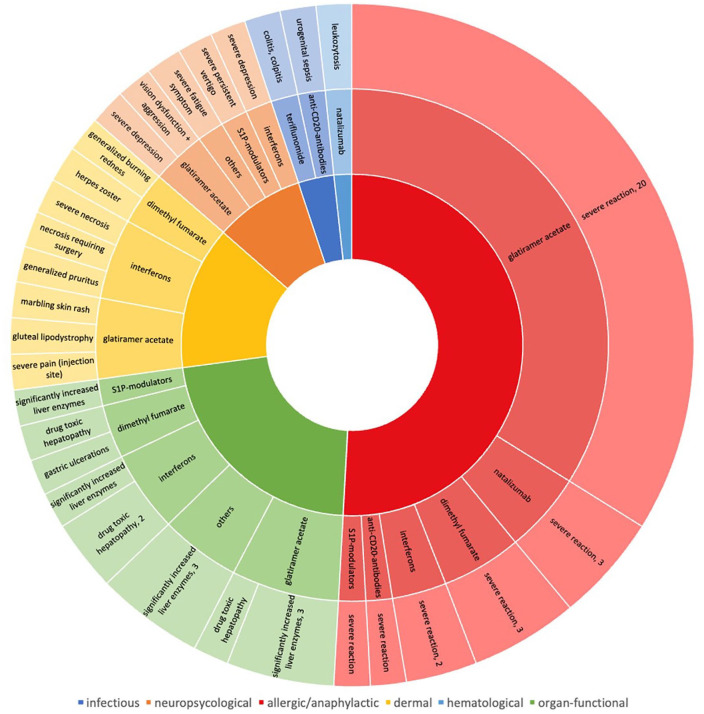
Adverse events with unusual severity. Proportional representation of adverse events with unusual severity (*n* = 59) in the various drug groups. Clusters across organs to improve comprehensibility (infectious, hematologic, neuropsychological, dermal, organ-functional, and allergic/anaphylactic). Multiple occurrences of an event are indicated by a number. CD, cluster of differentiation; S1P-modulators, sphingosine-1-phosphate receptor modulators.

The classification of recorded AEs revealed that 91.9% were categorized as common, 3.1% as known but unusually severe, and 1.7% as atypical. While most observed AEs aligned with the known and common AEs discussed with patients before treatment initiation, certain AEs exhibited unusual severity, prompting a distinct reporting. Figure 5 summarizes these AEs associated with DMT use, characterized by an unusual intensity or extent. Noteworthy instances include abscess surgery for interferon-induced necrosis, urosepsis associated with anti-CD20 antibodies, and severe depression linked to interferons and glatiramer acetate. Although described as common AEs associated with certain drugs (DMF, glatiramer acetate, interferons), 12 cases were reported to have unusually severe elevations of aspartate aminotransferase (AST, normal value 5–31 units/liter (U/l) in women and 5–35 U/l in men) and alanine aminotransferase (ALT, normal value <35 U/l in women and <50 U/l in men) to 5–10 times the normal range including toxic hepatopathy. A majority of unusually severe AEs related to anaphylaxis were linked to glatiramer acetate treatment. Collectively, allergic AEs to all DMTs contributed to approximately half (*n* = 30) of the unusually severe AEs in our study cohort.

**Figure 6.**
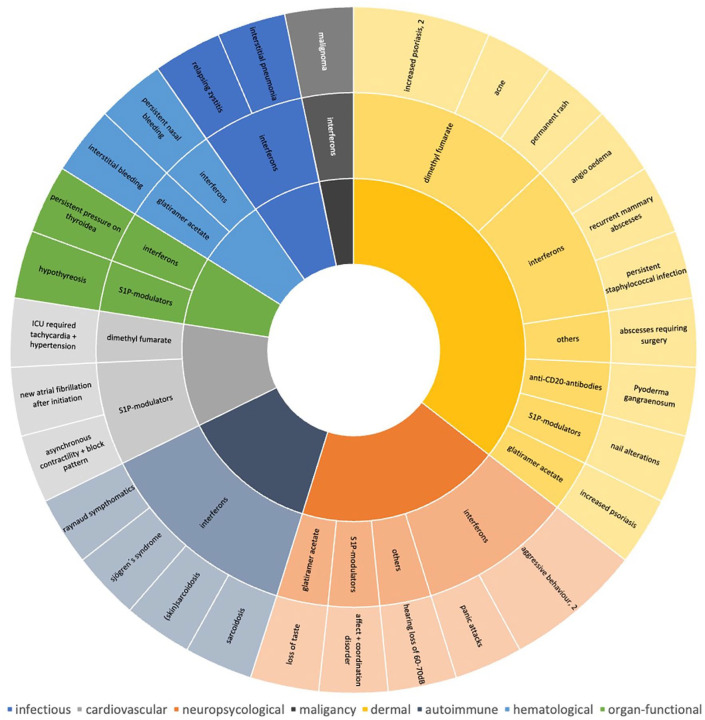
Adverse drug reactions with atypical manifestations. A proportional overview illustrating all adverse drug reactions with atypical manifestations (*n* = 31) not widely described in the current literature. Clustering across organs among drug groups for visualization (infectious, hematologic, organ-functional, cardiovascular, autoimmune, neuropsychological, dermal). CD, cluster of differentiation; dB, decibel; ICU, intensive care unit; S1P-modulators, sphingosine-1-phosphate receptor modulators.

A detailed analysis identified 31 atypical AEs not previously specified in corresponding medicinal product information; these are described in Table 3 and Figure 6. Atypical cutaneous side effects occurred most frequently, followed by neuropsychological and autoimmune AEs. Atypical side effects in the cardiovascular system, on the thyroid gland, related to hemostasis and infections, as well as malignancies occurred less frequently. Only 36% of these atypical AEs have been mentioned in previous publications available in PubMed. In contrast, when searching for AEs in the European-wide database EudraVigilance, we found 86% of atypical AEs previously reported. This underlines that significantly more side effects can be attributed to immunotherapies in real-world studies and large international databases than in published cases of side effects in literature.

**Table 3.** Summary of atypical adverse events not referenced in the corresponding medical product information. Additionally, case reports identified in the current literature (PubMed) and in EudraVigilance, an information system for recording potential adverse events, are listed.

Sex/age	MS form	Age at onset	Drug	Type of atypical adverse event	Treatment	Outcome	Number of previous cases	References (August 2023)	EudraVigilance (October 2023)
F/33	RRMS	26	pegIFN-β1a s.c.	Aggressive behavior	Conservative, change to DMF	TR with DMF			7 Cases with aggression
F/49	RRMS	39	IFN-β1a s.c.	Aggressive behavior	Conservative, change to GA	TR with GA			46 Cases with aggression
M/61	SPMS	44	IFN-β1a i.m.	Interstitial pneumonia with drumstick finger	Conservative, no further therapy	n.d.	CR: 1 Parenchyma hypercapillarization; 5 PAHT in 3 papers, 1 Broncholitis obliterans with organized pneumonia	Capobianco et al.,^18^ Fok et al.,^19^ Lerche et al.,^20^ Caravita et al.^21^	21 Cases with interstitial lung disease
F/45	RRMS	28	IFN-β1a s.c.	Recurrent mammary abscesses	Conservative, continuation with IFN-β1a s.c. after 3 years of therapy break	TR without further abscesses after restart of IFN-β1a s.c.			1 Case with breast inflammation
F/50	RRMS	37	IFN-β1a s.c.	Cutaneous sarcoidosis	Conservative, no further therapy	TR with no further therapy	CR: 2 Sarcoidosis with skin manifestation in 2 papers	Chakravarty et al.,^22^ Viana de Andrade et al.^23^	6 Cases with cutaneous sarcoidosis
F/50	RRMS	44	IFN-β1a s.c.	Sarcoidosis	Conservative, no further therapy	TR with no further therapy	CR: 2 Sarcoidosis with skin manifestation		6 Cases with cutaneous sarcoidosis
F/41	RRMS	27	IFN-β1b s.c.	Questionable triggering of Sjögren’s syndrome	Conservative, no further therapy	After 2 years diagnosis of pulmonal sarcoidosis with continuous Prednisolon 10 mg (pulmonal symptoms after try of reduction)	No previous reports		No previous reports
M/52	RRMS	25	IFN-β1b s.c.	Raynaud’s symptoms	Conservative, no further therapy	n.d.	CR: 1 Raynaud’s syndrome	Rot and Ledinek^24^	No previous reports
F/53	RRMS	43	IFN-β1a s.c.	Staphylococcal infection of the skin	Conservative with antibiotics and change to DMF	Improved skin infection after dermatological treatment			17 Cases with dermatitis
F/62	RRMS	52	IFN-β1a i.m.	Relapsing cystitis	Conservative with antibiotics and change to S1PM	TR with S1PM			2 Cases with hemorrhagic cystitis
F/39	RRMS	21	IFN-β1a i.m.	Panic attacks	Conservative with antidepressants, change to S1PM	TR with S1PM			85 Cases with panic attacks
F/45	RRMS	19	IFN-β1a i.m.	Malignant melanoma abdominal	Discontinuation of treatment	n.d.			Insufficient data
F/51	RRMS	37	IFN-β1a s.c.	Angio edema with chronic urticaria of uncertain etiology	Conservative with antihistamines	No further visits at our department	CR: 1 urticarial IgE-mediated reaction (IFN-β1b s.c.), 3 widespread urticaria (IFN-β1a i.m.)	Brown et al.,^25^ Guijarro et al.,^26^ Mazzeo et al.^27^	2 Cases with chronic urticaria
F/38	RRMS	25	IFN-β1a i.m.	Persistent epistaxis	Conservative, change to IFN-β1b	TR with IFN-β1b s.c.	No previous reports		No previous reports
F/42	RRMS	34	IFN-β1a i.m.	Persistent pressure on thyroidal	Conservative, change to DMF	TR with DMF	0, Different cases of thyroidal dysfunction (IFN-β1b s.c.)		No previous reports
F/47	RRMS	34	GA	Loss of taste, skin ulceration	Conservative, change to NAT	TR with NAT	0		15 Cases with ageusia, 57 cases with dysgeusia, 17 cases with skin ulcer
M/56	RRMS	51	GA	Increased psoriasis	Conservative, change to S1PM	Improved disease control	0		13 Cases with psoriasis
F/60	RRMS	42	GA	Intestinal bleeding	Conservative, change to IFN-β1a i.m.	TR with IFN-β1a i.m.	0		4 Cases with intestinal hemorrhage
M/71	SPMS	61	DMF	Tachycardia requiring intensive care and high blood pressure	Conservative, steroid pulse and fampridine since 5 years	n.d.	0		59 Cases with tachycardia
F/51	RRMS	40	DMF	Increased psoriasis	Conservative, change to NAT	Improvement of symptoms	0		86 Cases with psoriasis
F/47	RRMS	44	DMF	Increased psoriasis	Conservative, no further therapy	Improvement of symptoms	0		86 Cases with psoriasis
M/24	RRMS	21	DMF	Acne	Conservative, no further therapy	Improved skin	0		33 Cases with acne
F/72	PPMS	60	S1PM (Fingolimod)	Nail change (dermatologically clarified; no fungus/bacteria), tongue swelling, hemorrhagic tracheobronchitis	Conservative, change to mitoxantrone	Persisting hemoptysis after discontinuation, TR of nail alteration	0		1 Case with nail change, 2 cases with tracheal disorder
F/46	RRMS	42	S1PM (Fingolimod)	Affective disorder and coordination disorder	Conservative, change to GA	Improvement of symptoms	CR: 1 affective disorder	Fitzpatrick et al.^28^	12 Cases with affective disorder
F/52	RRMS	41	S1PM (Fingolimod)	New-onset bradycardic atrial fibrillation with arrhythmia absoluta at initial administration	Conservative, high dose heparin with dalteparin 5000 IU, change to NAT	Spontaneous conversion to sinus rhythm with no need of further diagnostics	CR: 2 (P)AF at initial administration, 1 arrhythmia absoluta at initial administration	Rolf et al.,^29^ Brown et al.,^30^ Laroni et al.^31^	106 Cases with atrial fibrillation
F/52	RRMS	40	S1PM (Fingolimod)	Hypothyroidism (Exacerbation of existing latent hypothyroidism with neg. TPO-AK)	Conservative, initiation with L-thyroxine and change to NAT	TR of thyroid hormones and stop of L-thyroxine, normal thyroidea in sonography	CR: 1 hypothyroidism	Flores et al.^32^	24 Cases with hypothyroidism
M/54	RRMS	50	S1PM (Fingolimod)	Asynchronous contractility and block pattern	Monitoring on ward for several days with spontaneous remission, change to NAT	Spontaneous conversion to sinus rhythm with no need of further diagnostics	Ischemia and reperfusion-induced AVN rhythmic disturbance in in vitro rat model	Egom et al.^33^	102 Cases with atrioventricular block
F/24	RRMS	19	aCD20 (Ocrelizumab)	Pyoderma gangrenosum gluteal/genital	Multiple surgical wound care; currently in dermatology care with involvement of pain service (first OP 07/20, last OP 08/21)	In continuing treatment, last dose 06/21	CR: 2 vulvovaginal pyoderma gangrenosum after ocrelizumab treatment for MS	Klumpp et al.,^34^ Breneman et al.^35^	9 Cases (2 fatal)
M/56	RRMS	52	Mitoxantrone	Pantonal hearing loss of 60–70 dB without remission	Intratympanic injection therapy	No further visits at our department	Mild hearing loss in 6 patients treated with mitoxantrone and cytarabine for acute leukemia	Solary et al.^36^	1 Case with permanent deafness
F/41	RRMS	27	Azathioprine	Abscesses requiring surgery	Surgical treatment and change to mitoxantrone	TR without further abscesses	CR: 2 with intraabdominal abscesses	Northfield and Roberts^37^	2 Cases with abscess drainage
F/44	RRMS	37	DMF	Permanent skin rash	Conservative, change to IFN-β1a	TR after discontinuation	CR: 1 erythema nodosum	Algahtani et al.^38^	

aCD20, anti-CD20-antibodies; AVN, atrioventricular node; CD20, cluster of differentiation 20; CR, case report; dB, decibel; DMF, dimethyl fumarate; F, female; GA, glatiramer acetate; IFN-β1a/b, interferon beta 1a/b; IgE, immunoglobulin E; i.m., intramuscular; IU, international unit; M, male; MS, multiple sclerosis; NAT, natalizumab; n.d., not determined; OP, operation; (P)AF, (paroxysmal) atrial fibrillation; PAHT, pulmonary arterial hypertension; PPMS, primary progressive multiple sclerosis; RRMS, relapsing-remitting multiple sclerosis; S1PM, sphingosine-1-phosphate receptor modulators; s.c., subcutaneous; SPMS, secondary progressive multiple sclerosis; TPO-AK, thyroid peroxidase antibodies; TR, total remission.

## Discussion

In a large real-world cohort with MS and related diseases, we studied clinical characteristics, therapy sequencing, and the occurrence of unusually severe and atypical AEs upon DMTs. The findings that 51.2% of treatments were unaccompanied by reported AEs while 3.1% resulted in unusually severe and 1.7% in atypical AEs, together with their specificities offer valuable insights into the treatment outcomes and safety profiles of diverse DMTs in a real-world setting. Notably, the systematic and comprehensive approach of our investigation goes beyond previous efforts that mainly focus on common side effects such as infections^39^ or only focus on specific single drugs.^40^

Initiation of therapy with interferons, glatiramer acetate, and DMF is consistent with patterns in other real-world cohorts,^41^ with higher efficacy DMTs such as S1P modulators, anti-CD20 antibodies, and natalizumab constituting 22% of therapy cases. The implementation of high efficacy DMTs as second-line treatments reflects an escalating therapy concept which currently gradually turns into more aggressive treatment from the beginning.^2^ While no clear sex-based differences in DMT response were documented in a previous review,^42^ our study reveals sex disparities in treatment patterns. Males exhibit a significantly higher proportion of high-efficacy DMTs as second or third treatments, suggesting nuanced considerations in treatment decision-making influenced by sex-specific factors such as family planning.

The average duration of DMT use of 4.4 ± 0.1 years slightly exceeds previous studies,^43^ indicating a potential trend toward extended treatment periods in our cohort. Approximately 50% of patients switched DMTs, with DMF and S1P modulators being prominent in subsequent therapies. This underscores the dynamic nature of treatment strategies and the need for adaptability in managing MS disease activity. Lack of efficiency emerged as the primary cause for treatment adjustment, followed by AEs. This finding is in line with comparable real-world studies,^44^ emphasizing the significance of treatment effectiveness and safety considerations in clinical decision-making.

The probability of AE occurrence was similar among all DMTs, with AEs generally occurring in less than 50% of cases. Importantly, more than half of the cases allowed for the continuation of DMTs despite AEs, highlighting the tolerability of certain treatments, notably anti-CD20 antibodies and natalizumab. The majority of recorded AEs align with known probabilities of occurrence, as indicated by regulatory authorities. This alignment emphasizes the reliability of safety data in informing treatment decisions. Notably, a subset of AEs, particularly allergic reactions, exhibited unusual severity, representing 3.1% of common AEs. This underscores the importance of closely monitoring and addressing severe manifestations, even for events considered common.

Atypical AEs, not discovered in the registration trials or in medicinal product information as checked by the Committee for Medicinal Products for Human Use (CHMP) and Bundesinstitut für Arzneimittel und Medizinprodukte (Federal Institute for Drugs and Medical Devices) (BfArM) were rare, emphasizing the value of real-world cohort studies. A case of pyoderma gangrenosum secondary to ocrelizumab treatment in our cohort^34^ matched with the one reported individual from another center with this complication.^35^ Long-term dermatological treatment and the use of cyclosporine resulted in slow healing in the case observed at our center. Other examples are the occurrence of tachycardia in patients treated with DMF, not yet published, but found in our cohort and reported in the EudraVigilance database, and epistaxis upon IFN-β, neither reported in the EudraVigilance database nor published in PubMed, which ceased after change of treatment.

While only 36% of atypical side effects have been previously reported in articles in PubMed, reports of around 86% of these atypical AEs in the EudraVigilance database support that the occurrence of these events is most likely not a random association.

This study has its limitations due to the rather long recruiting period with different market-launch time points of DMTs restricting interpretations on proportional distribution among DMTs. In addition, limitations occurred in the evaluation of treatment periods, especially for patients who contributed multiple data points and who had been treated at other institutions in the past. Due to incomplete information from the patients or medical documentation, these data could not be included in the analysis. Furthermore, the data sources relied predominantly on subjective assessments of AEs. Standardized tools for AE assessment or predefined questions were not used in daily routine. Additionally, the weaker evidence for causal associations between drug exposure and observed AEs remains a limitation in observational studies without comparable control groups. Another limitation is that the study did not include a calculation of the sample size selected for this study.

## Conclusion

Overall, we conclude from this real-world cohort study that, despite the occurrence of AEs in approximately half of the patients—a common finding—DMTs are generally well-tolerated and safe for MS patients and individuals with related conditions, consistent with findings from a recent Cochrane Review.^45^ Interestingly, we observed sex differences in treatment choices with females receiving a lower proportion of high-efficacy DMTs that we interpret as a consequence of family planning considerations. Although greater uncertainty regarding causality must be acknowledged, the observation of a smaller number of atypical AEs extends the spectrum of AEs associated with DMTs and demonstrates the value of real-world investigations in offering insights into the long-term safety of DMTs, particularly for rare events.

## Supplemental Material

sj-pdf-1-tan-10.1177_17562864251320206 – Supplemental material for Atypical adverse events in a real-world study of long-term immunomodulation for multiple sclerosis and neuromyelitis optica spectrum disorder Supplemental material, sj-pdf-1-tan-10.1177_17562864251320206 for Atypical adverse events in a real-world study of long-term immunomodulation for multiple sclerosis and neuromyelitis optica spectrum disorder by Amelie Kirschbaum, Felix Luessi, Arda Civelek, Stefan Bittner, Johannes Piepgras and Frauke Zipp in Therapeutic Advances in Neurological Disorders

sj-pdf-2-tan-10.1177_17562864251320206 – Supplemental material for Atypical adverse events in a real-world study of long-term immunomodulation for multiple sclerosis and neuromyelitis optica spectrum disorder Supplemental material, sj-pdf-2-tan-10.1177_17562864251320206 for Atypical adverse events in a real-world study of long-term immunomodulation for multiple sclerosis and neuromyelitis optica spectrum disorder by Amelie Kirschbaum, Felix Luessi, Arda Civelek, Stefan Bittner, Johannes Piepgras and Frauke Zipp in Therapeutic Advances in Neurological Disorders

sj-pptx-3-tan-10.1177_17562864251320206 – Supplemental material for Atypical adverse events in a real-world study of long-term immunomodulation for multiple sclerosis and neuromyelitis optica spectrum disorder Supplemental material, sj-pptx-3-tan-10.1177_17562864251320206 for Atypical adverse events in a real-world study of long-term immunomodulation for multiple sclerosis and neuromyelitis optica spectrum disorder by Amelie Kirschbaum, Felix Luessi, Arda Civelek, Stefan Bittner, Johannes Piepgras and Frauke Zipp in Therapeutic Advances in Neurological Disorders

## Appendix

### Abbreviations

AE adverse event

AL alemtuzumab

ALT alanine aminotransferase

AST aspartate aminotransferase

BfArM Bundesinstitut für Arzneimittel und Medizinprodukte (Federal Institute for Drugs and Medical Devices)

CD20 cluster of differentiation 20

CHMP Committee for Medicinal Products for Human Use

CIS clinically isolated syndrome

CIS/RIS clinically/radiologically isolated syndrome

DMT disease-modifying therapy

BG-12/DMF dimethyl fumarate

EMA European Medicines Agency

FDA Food and Drug Administration

HED high-efficacy drugs

ICD-10 International Classification of Diseases, 10th Revision

ICU intensive care unit

IFN interferon

IFN-β1a/b interferon beta 1a/b

IQR interquartile range

JCV John Cunningham virus

MS multiple sclerosis

MTX methotrexate

NMOSD neuromyelitis optica spectrum disorder

ON optic neuritis

pegIFN-β1b pegylated interferon beta 1b

PPMS primary progressive multiple sclerosis

RIS radiologically isolated syndrome

RRMS relapsing-remitting multiple sclerosis

RTX rituximab

S1P sphingosine-1-phosphate

S1PM sphingosine-1-phosphate receptor modulators

SPMS secondary progressive multiple sclerosis

## Declarations

**Ethics approval and consent to participate:** This study is covered by the State Hospital Act §36 and §37 and thus did not require written consent, according to the local ethics committee (Landesärztekammer Rheinland-Pfalz).

**Consent for publication:** Not applicable.

**Competing interests:** This study is a part of the doctoral thesis (MD) of AK at the University Medical Center of the Johannes Gutenberg University Mainz, Germany. F.L. received consultancy fees from Roche and support with travel cost from Teva. S.B. has received honoraria from Biogen Idec, Bristol Meyer Squibbs, Merck Healthcare, Novartis, Roche, Sanofi Genzyme, and TEVA. His research is funded by Deutsche Forschungsgemeinschaft (DFG), Hertie Foundation, and the Hermann and Lilly-Schilling Foundation. F.Z. has recently received research grants and/or consultation funds from Biogen, Ministry of Education and Research (BMBF), Bristol-Meyers-Squibb, Celgene, German Research Foundation (DFG), Janssen, Max-Planck-Society (MPG), Merck Serono, Novartis, Progressive MS Alliance (PMSA), Roche, Sanofi Genzyme, and Sandoz. A.K. and J.P. report no competing interests.

**Availability of data and materials:** The raw data used in the preparation of the figures and tables will be shared in anonymized format on request of a qualified investigator to the corresponding author for purposes of replicating procedures and results.
