# Supplementary material for: Atypical adverse events in a real-world study of long-term immunomodulation for multiple sclerosis and neuromyelitis optica spectrum disorder
Source: Ther Adv Neurol Disord. 2025 Apr 4;18:17562864251320206. doi: 10.1177/17562864251320206 (PMC12032468; doi:10.1177/17562864251320206)
Supplement: sj-pdf-2-tan-10.1177_17562864251320206 – Supplemental material for Atypical adverse events in a real-world study of long-term immunomodulation for multiple sclerosis and neuromyelitis optica spectrum disorder [file sj-pdf-2-tan-10.1177_17562864251320206.pdf]

Supplementary Table 1

Sex/age	MS form	age at onset	drug	type of sAE	treatment	outcome	frequency (according to prescribing information)
f/69	RRMS	52	GA	sAE severe allergic reaction	conservative, change to Mitoxantrone	TR, no further allergic reactions	Allergic reactions are uncommon and are not listed among adverse reactions with an incidence >2% in clinical trials, their frequency cannot be estimated
f/25	RRMS	15	GA	sAE severe allergic reaction	conservative, change to IFN	TR, no further allergic reactions	Allergic reactions are uncommon and are not listed among adverse reactions with an incidence >2% in clinical trials, their frequency cannot be estimated
f/48	RRMS	48	IFN-ß1a s.c.	sAE severe necrosis	conservative, change to Mitoxantrone	TR, no further necrosis	Injection site reactions are very common, yet necroses are rather occasional.
m/56	RRMS	n.d.	GA	sAE severe allergic reaction	conservative, change to IFN	TR, no further allergic reactions	Allergic reactions are uncommon and are not listed among adverse reactions with an incidence >2% in clinical trials, their frequency cannot be estimated
m/49	RRMS	n.d.	GA	sAE severe allergic reaction	conservative, change to IFN	TR, no further allergic reactions	Allergic reactions are uncommon and are not listed among adverse reactions with an incidence >2% in clinical trials, their frequency cannot be estimated
f/44	RRMS	39	DMF	sAE drug toxic hepathopathy	conservative, change to GA	TR	rare
m/49	RRMS	39	others (Azathioprin)	sAE severe fatigue symptom	conservative, no further therapy documented	no further visits at our department	The incidence of fatigue specifically is not always clearly defined in clinical trials or patient reports, but it is often described as a common non-specific symptom associated with the use of azathioprine. Fatigue is estimated to occur in 10-20% of patients, depending on the study and patient cohort.
f/41	RRMS	26	GA	sAE severe allergic reaction	conservative, change to IFN	TR, aAE under IFN: Sjögren-Syndrome	Allergic reactions are uncommon and are not listed among adverse reactions with an incidence >2% in clinical trials, their frequency cannot be estimated. The emergence of Sjögren-Syndrome due to DMF has so far not been published in the literature.
f/47	RRMS	40	IFN-ß1a s.c.	sAE severe allergic reaction	conservative, no further therapy documented	no further visits at our department	Uncommon, affecting 1 in 1000 patients.
f/52	RRMS	26	IFN-ß1a s.c.	sAE necrosis requiring surgery	External surgical necrosis removal, change to IFN	renewed necrosis under IFN, change to GA. After sAE with severe allergic reaction change to Mitoxantrone. After sAE with significantly increased liver enzymes (5x) change to NAT	Injection site reactions are very common, yet necroses are rather occasional.
m/51	RRMS	41	GA	sAE vision dysfunction and aggressive behavior	conservative, no further therapy documented	no further visits at our department	Visual disturbances were observed in 3 patients in controlled clinical trials (n: 563), there is no cases of particular aggresion as adverse event in the literature, as such, ist frequency cannot be estimated
f/51	RRMS	40	GA	sAE severe allergic reaction	conservative, change to DMF	TR, aAE under DMF: increased psoriasis	Allergic reactions are uncommon and are not listed among adverse reactions with an incidence >2% in clinical trials, their frequency cannot be estimated
f/56	RRMS	n.d.	GA	sAE gluteal lipodystrophy	conservative, no further therapy documented	no further visits at our department	Not previously described in the literature.
f/48	RRMS	39	GA	sAE severe allergic reaction	conservative, change to IFN	TR, sAE under IFN: severe allergic reaction, TR without further allergic reactions after therapy stop	Allergic reactions are uncommon and are not listed among adverse reactions with an incidence >2% in clinical trials, their frequency cannot be estimated. The frequeny of allergic reactions through application of IFN is also not known.
f/45	RRMS	39	GA	sAE severe allergic reaction	conservative, change to Mitoxantrone	TR, no further allergic reactions	Allergic reactions are uncommon and are not listed among adverse reactions with an incidence >2% in clinical trials, their frequency cannot be estimated
f/39	RRMS	n.d.	GA	sAE severe allergic reaction	conservative, change to IFN	TR, no further allergic reactions	Allergic reactions are uncommon and are not listed among adverse reactions with an incidence >2% in clinical trials, their frequency cannot be estimated
f/37	RRMS	n.d.	IFN-ß1b s.c.	sAE drug toxic hepathopathy	conservative, change to aCD20 (Ocrelizumab)	TR under external therapy with therapy break, therapy initiation with aCD20	According to LiverTox, it is linked to rare instances of clinically apparent liver injury with jaundice
f/34	SPMS	16	IFN-ß1a i.m.	sAE severe depression	conservative, change to GA	TR, further depression under GA (sAE)	very common (up to 1 in 10 patients)
f/58	RRMS	39	GA	sAE severe allergic reaction	conservative, change to S1PM (Fingolimod)	TR, no further allergic reactions, lymphopenia during therapy with S1PM	Allergic reactions are uncommon and are not listed among adverse reactions with an incidence >2% in clinical trials, their frequency cannot be estimated
f/42	RRMS	33	GA	sAE marbling skin rash	conservative, change to IFN	TR	
f/40	RRMS	34	IFN-ß1a s.c.	sAE significantly increased liver enzymes (7x)	conservative, change to GA	TR	According to LiverTox, it is linked to rare instances of clinically apparent liver injury with jaundice
f/50	RRMS	41	GA	sAE significantly increased liver enzymes (5x)	conservative, no further therapy documented	no further visits at our department	According to LiverTox, in large randomized controlled trials of glatiramer acetate in patients with multiple sclerosis, serum ALT elevations above 3 times ULN were reported in 7% of glatiramer compared to 3% of placebo recipients. The enzyme elevations were usually transient and not associated with symptoms or jaundice, requiring drug discontinuation in less than 1% of patients.
f/59	RRMS	n.d.	IFN-ß1a s.c.	sAE drug toxic hepathopathy	conservative, change to GA	TR, then further significantly increased liver enzymes under GA (sAE), no further visits at our department	According to LiverTox, it is linked to rare instances of clinically apparent liver injury with jaundice
m/48	RRMS	43	NAT	sAE severe allergic reaction	conservative, change to aCD20 (Ocrelizumab)	TR, no further allergic reactions	The overall incidence of allergic reactions, including hypersensitivity reactions, is estimated to be around 1-2% of patients receiving natalizumab.
m/71	RRMS	56	IFN-ß1a i.m.	sAE severe allergic reaction	conservative, no further therapy documented	TR, no further allergic reactions	Uncommon, affecting 1 in 1000 patients.
m/29	RRMS	28	aCD20 (Ocrelizuma	sAE severe allergic reaction	conservative, no further therapy documented	TR, no further allergic reactions	No hypersensitivity reactions to OCREVUS were reported in the controlled clinical trials.
m/52	RRMS	42	DMF	sAE severe allergic reaction	conservative, change to DMF	TR, no further allergic reactions	The frequency of severe allergic reactions cannot be estimated from the available data
f/61	RRMS	45	DMF	sAE severe allergic reaction	conservative, change to S1PM (Fingolimod)	TR, no further allergic reactions	The frequency of severe allergic reactions cannot be estimated from the available data
m/56	RRMS	32	GA	sAE severe allergic reaction	conservative, change to IFN	TR, no further allergic reactions	Allergic reactions are uncommon and are not listed among adverse reactions with an incidence >2% in clinical trials, their frequency cannot be estimated
f/59	RRMS	51	DMF	sAE generalized burning redness	conservative, change to GA	TR, no further allergic reactions	common
f/43	RRMS	21	GA	sAE severe allergic reaction	conservative, no further therapy documented	TR, no further allergic reactions	Allergic reactions are uncommon and are not listed among adverse reactions with an incidence >2% in clinical trials, their frequency cannot be estimated
f/36	RRMS	28	NAT	sAE severe allergic reaction	conservative, change to S1PM (Fingolimod)	TR, no further allergic reactions	The overall incidence of allergic reactions, including hypersensitivity reactions, is estimated to be around 1-2% of patients receiving natalizumab.
f/69	RRMS	47	GA	sAE severe allergic reaction	conservative, change to IFN	TR, no further allergic reactions	Allergic reactions are uncommon and are not listed among adverse reactions with an incidence >2% in clinical trials, their frequency cannot be estimated
f/46	RRMS	35	DMF	sAE gastric ulcerations	conservative therapy with pantoprazol, change to TER	TR	The frequency of gastric ulcerations cannot be estimated, these are not listed among adverse effects observed in clinical trials.
f/57	RRMS	41	GA	sAE significantly increased liver enzymes (5x)	conservative, no further therapy documented	no further visits at our department	According to LiverTox, in large randomized controlled trials of glatiramer acetate in patients with multiple sclerosis, serum ALT elevations above 3 times ULN were reported in 7% of glatiramer compared to 3% of placebo recipients. The enzyme elevations were usually transient and not associated with symptoms or jaundice, requiring drug discontinuation in less than 1% of patients.
f/28	RRMS	21	GA	sAE severe pain (injection site)	conservative with pain medication, change to DMF	TR	common
f/69	RRMS	53	GA	sAE severe allergic reaction	conservative, no further therapy documented	TR, no further allergic reactions	Allergic reactions are uncommon and are not listed among adverse reactions with an incidence >2% in clinical trials, their frequency cannot be estimated

f/46	RRMS	29	GA	sAE severe allergic reaction	conservative, change to Mitoxantron	TR, no further allergic reactions	Allergic reactions are uncommon and are not listed among adverse reactions with an incidence >2% in clinical trials, their frequency cannot be estimated
f/58	RRMS	26	GA	sAE severe allergic reaction	conservative no further therapy documented	TR, no further allergic reactions	Allergic reactions are uncommon and are not listed among adverse reactions with an incidence >2% in clinical trials, their frequency cannot be estimated
f/60	SPMS	51	DMF	sAE herpes zoster	conservative external therapy with aciclovir, change to S1PM (Fingolimod)	external therapy with therapy break, no residuals at therapy initiation with S1PM (Fingolimod)	There are cases with herpes infections reported, where ist frequency cannot be estimated.
m/42	RRMS	34	S1PM (Fingolimod)	sAE severe allergic reaction	conservative, change to DMF	TR, no further allergic reactions	The overall incidence of allergic reactions with fingolimod is low. In clinical trials, less than 1% of patients experienced significant allergic
f/60	RRMS	n.d.	NAT	sAE severe leukozytosis	conservative, change to IFN	TR	The incidence of leukocytosis in patients treated with natalizumab is estimated to be around 2-5%. The white blood cell count increase is typically modest and is usually not associated with significant clinical issues.
f/42	RRMS	34	DMF	sAE severe allergic reaction	conservative, change to S1PM (Fingolimod)	TR, no further allergic reactions	The frequency of severe allergic reactions cannot be estimated from the available data
f/40	RRMS	35	S1PM (Fingolimod)	sAE significantly increased liver enzymes (5x)	conservative, change to CdA	TR	Most liver enzyme elevations are mild to moderate and asymptomatic. Severe liver toxicity (marked by significantly elevated liver enzymes) is much less common, with rates of severe elevations being typically less than 1% of patients.
f/44	RRMS	33	GA	sAE severe allergic reaction	conservative, change to NAT	TR, no further allergic reactions	Allergic reactions are uncommon and are not listed among adverse reactions with an incidence >2% in clinical trials, their frequency cannot be estimated
f/52	RRMS	41	GA	sAE severe allergic reaction	conservative, change to DMF	TR, no further allergic reactions	Allergic reactions are uncommon and are not listed among adverse reactions with an incidence >2% in clinical trials, their frequency cannot be estimated
f/34	RRMS	22	aCD20 (Ocrelizuma	sAE urogential sepsis	conservative, no further therapies	no further visits at our department	Urinary tract infections are not among the infections and infestations observed in the pooled date from trials WA21092 & WA21093 as well WA25046
f/60	RRMS	55	others (Mitoxantrone	sAE significantly increased liver enzymes (5x)	conservative, no further therapies	TR	Mild to moderate increases in liver enzymes are common, but severe liver toxicity (including hepatotoxicity) is less frequent. The incidence of severe liver enzyme elevations is generally lower, estimated to be less than 5% of patients.
f/59	RRMS	54	GA	sAE significantly increased liver enzymes (5x)	conservative, change to S1PM (Fingolimod)	TR	According to LiverTox, in large randomized controlled trials of glatiramer acetate in patients with multiple sclerosis, serum ALT elevations above 3 times ULN were reported in 7% of glatiramer compared to 3% of placebo recipients. The enzyme elevations were usually transient and not associated with symptoms or jaundice, requiring drug discontinuation in less than 1% of patients.
f/55	RRMS	n.d.	others (Mitoxantrone	sAE significantly increased liver enzymes (6x)	conservative, change to S1PM (Fingolimod)	TR	Mild to moderate increases in liver enzymes are common, but severe liver toxicity (including hepatotoxicity) is less frequent. The incidence of severe liver enzyme elevations is generally lower, estimated to be less than 5% of patients.
f/58	RRMS	51	GA	sAE severe allergic reaction	conservative, change to Mitoxantrone	TR, no further allergic reactions	Allergic reactions are uncommon and are not listed among adverse reactions with an incidence >2% in clinical trials, their frequency cannot be estimated
f/36	RRMS	25	NAT	sAE severe allergic reaction	conservative, change to NAT	TR, no further allergic reactions	The overall incidence of allergic reactions, including hypersensitivity reactions, is estimated to be around 1-2% of patients receiving
f/53	RRMS	43	S1PM (Fingolimod)	sAE severe persistent vertigo	conservative, change to IFN	TR, aAE under IFN: persistent staphylococcal infection	The incidence of vertigo specifically as an adverse effect of fingolimod is low, occurring in approximately 1-2% of patients based on clinical trial data and post-marketing reports.
f/41	RRMS	37	TER	sAE colitis	conservative, no further therapies documented	no further visits at our department	Exact figures for the incidence of colitis are not commonly listed in general references, but it is considered to be rare compared to more common gastrointestinal issues such as diarrhea.
