# Supplementary material for: Atypical adverse events in a real-world study of long-term immunomodulation for multiple sclerosis and neuromyelitis optica spectrum disorder
Source: Ther Adv Neurol Disord. 2025 Apr 4;18:17562864251320206. doi: 10.1177/17562864251320206 (PMC12032468; doi:10.1177/17562864251320206)
Supplement: sj-pptx-3-tan-10.1177_17562864251320206 – Supplemental material for Atypical adverse events in a real-world study of long-term immunomodulation for multiple sclerosis and neuromyelitis optica spectrum disorder [file sj-pptx-3-tan-10.1177_17562864251320206.pptx]

## Slide 1
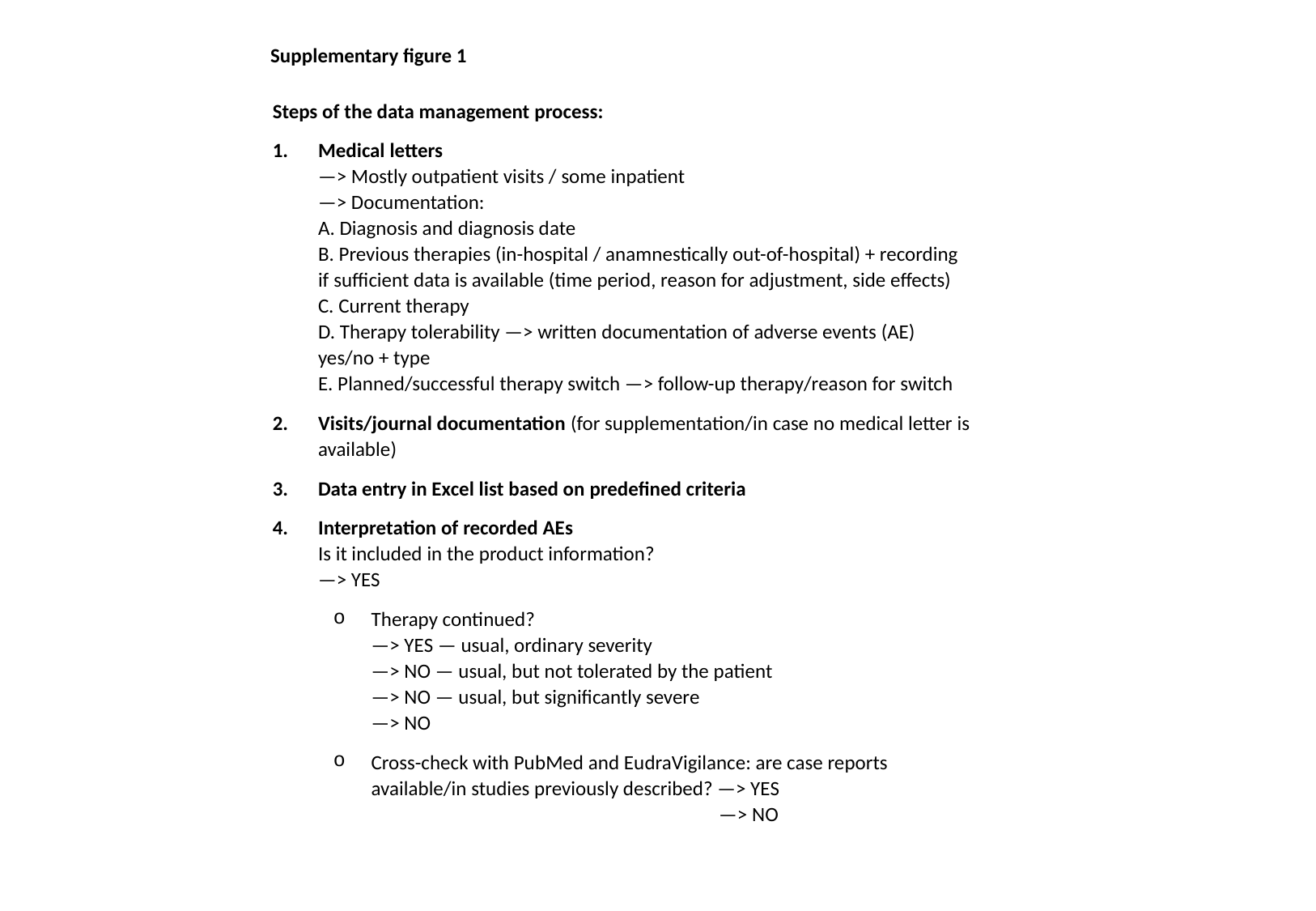

Supplementary figure 1
Steps of the data management process:
Medical letters
—> Mostly outpatient visits / some inpatient
—> Documentation:
A. Diagnosis and diagnosis date
B. Previous therapies (in-hospital / anamnestically out-of-hospital) + recording if sufficient data is available (time period, reason for adjustment, side effects)
C. Current therapy
D. Therapy tolerability —> written documentation of adverse events (AE) yes/no + type
E. Planned/successful therapy switch —> follow-up therapy/reason for switch
Visits/journal documentation (for supplementation/in case no medical letter is available)
Data entry in Excel list based on predefined criteria
Interpretation of recorded AEs
Is it included in the product information?
—> YES
Therapy continued?
—> YES — usual, ordinary severity
—> NO — usual, but not tolerated by the patient
—> NO — usual, but significantly severe
—> NO
Cross-check with PubMed and EudraVigilance: are case reports available/in studies previously described? —> YES
 —> NO
